# Post-discharge use of opioids, psychostimulants, and treatment medications following residential opioid discontinuation with NET Device™ monotherapy

**DOI:** 10.3389/fpsyt.2025.1627267

**Published:** 2025-10-15

**Authors:** Mark K. Greenwald, Cynthia L. Arfken, Joe R. Winston

**Affiliations:** ^1^ Department of Psychiatry and Behavioral Neurosciences, Wayne State University School of Medicine, Detroit, MI, United States; ^2^ NET Recovery Corp.^™^(NRC), Wilmington, DE, United States

**Keywords:** NET Device, tACS, opioid, psychostimulant, polysubstance, medication, MOUD

## Abstract

**Background:**

Medical devices offer an established therapeutic approach for managing the transition from polysubstance use to abstinence, but little is known about longer-term outcomes.

**Aim:**

Determine opioid and psychostimulant use over 12 weeks post-discharge following residential use of the NET Device™ or sham among participants with opioid use disorder (OUD).

**Design:**

Twelve-week observation following randomized controlled trial of active NET Device (n = 53) versus sham (n = 55) as monotherapy during residential opioid discontinuation in 103 participants who completed >1 post-discharge interview (95% follow-up rate).

**Setting and participants:**

Adults with OUD (50.0% with psychostimulant use) recruited at admission from 4 residential addiction treatment facilities in Kentucky.

**Measures:**

Percentage of days using illicit opioids and psychostimulants as well as medications for OUD (MOUD) from weekly timeline follow-back interviews.

**Results:**

Active and sham device groups reported similar rates of MOUD use and illicit opioid and psychostimulant use. In a planned secondary analysis, participants who self-administered active stimulation >24 hours (n = 23) compared to active <24 hours (n = 25), sham >24 hours (n = 21), and sham <24 hours (n = 34) reported significantly (p <.05) lower percentages of days using opioids (1.4% vs 7.4%, 6.8%, and 4.4% respectively) and psychostimulants (1.3% vs 4.1%, 6.7%, and 4.3% respectively) and MOUD (1.2% vs 20.7%, 11.3%, and 16.7% respectively).

**Conclusions:**

The randomized groups did not differ on outcomes. However, participants who self-administered active NET Device stimulation >24 hours reported significantly fewer post-discharge days of opioid or psychostimulant use than those who self-administered active <24 hours or sham, but these findings need to be replicated.

Clinical Trial Registration: ClinicalTrials.gov, identifier NCT04916600.

## Introduction

High rates of nonmedical/illicit opioid use and opioid use disorder (OUD) have adversely impacted societal, health and economic welfare across North America (1–6). In recent years, these problems have been exacerbated by exposure to synthetic opioids and psychostimulants (7–13). The unprecedented burden of opioid-related overdose deaths (14) and emergency department visits (15, 16) has motivated assertive and widespread initiatives to combat this crisis.

Despite this urgency, OUD treatment involvement in the US remains low. In 2021, about 2.5 million adults had past-year OUD, yet only 35.6% received any past-year treatment (17) and, due to systemic barriers (18–21), use of medications for OUD (MOUD) was only 22.2% (17). There is sociodemographic variation in who receives MOUD: those more likely to receive MOUD have more severe past-year OUD, receive treatment via telehealth, live in metropolitan areas, report family income <$50,000, are male and non-Hispanic White (17). Moreover, a sizable – but not yet well-estimated – proportion of patients entering OUD treatment express reservations about MOUD (e.g. side effects, inconvenience, stigma, expected long duration of treatment) and express curiosity about non-medication interventions for transitioning to longer-term abstinence (22–27), although presently such options are sparse. This gap between the desire for non-MOUD treatment and available alternatives could lead many OUD patients to avoid or drop out of otherwise lifesaving treatment.

Medical devices offer an emerging alternative therapeutic approach that could be useful in managing the transition from illicit opioid use to abstinence (28). To date, developmental efforts have focused on devices that can attenuate withdrawal discomfort during residential opioid discontinuation (29, 30). However, little is known about what happens post-discharge. NeuroElectric Therapy™ (NET^®^) is an FDA-cleared transcranial alternating current stimulation (tACS) neurostimulation modality for rapidly attenuating opioid withdrawal symptoms in persons with OUD, with and without comorbid polysubstance use, undergoing opioid discontinuation (31).

Earlier-generation versions of this device were studied in the inpatient setting as a monotherapy for medication-free discontinuation from substance use (32–35). After inpatient NET use, completers in those studies who could be followed post-discharge (49 out of 116 participants) underwent drug testing. Approximately 85% of them tested negative for illicit opioids, methadone, and buprenorphine and 98% tested negative for cocaine and methamphetamines (NET Recovery Corp., unpublished data). However, attrition was high, and participants knew the treatment they had received.

To provide more compelling data, we designed (36) and conducted a randomized, sham-controlled, blinded clinical trial that showed randomization to the active NET Device led to significant decreases in opioid withdrawal severity during residential opioid discontinuation (31). However, that report did not answer what happened to the participants after they left the residential treatment facility. This trial also prospectively planned a 12-week post-discharge follow-up period to measure longer-term outcomes.

For this observational study, we hypothesized that the group randomized to active stimulation during the residential period would report less-frequent use of illicit opioids and psychostimulants without use of MOUD compared to sham stimulation during the post-discharge period. The purpose of including non-use of MOUD in our outcome definition is to provide a rigorous and more conservative estimate of NET Device efficacy as a monotherapy. In prior open-label studies, participants have varied widely in the duration of their device stimulation, with some indication that extended use might offer greater benefit (NET Recovery Corp., unpublished data). Therefore, a secondary hypothesis was that participants who self-administered active stimulation longer would report greater reductions in opioid and psychostimulant use without the assistance of MOUD compared to other participants.

## Methods

### Study design

This was a 12-week prospective, observational period following a clinical trial (NCT04916600) described elsewhere (36). Briefly, the clinical trial was a double-blind, randomized, stratified by sex, single-site trial at four facilities to compare whether active device stimulation led to greater reduction in withdrawal symptom severity during residential opioid discontinuation (31).

The trial was originally designed and powered to detect an effect of active versus sham device use on post-discharge substance use as the primary efficacy endpoint. However, following FDA discussion, it was decided to seek device clearance through the 510(k) pathway for opioid withdrawal reduction during the residential period (37). Although the clinical trial was limited to the first hour of the intervention, the post-discharge prospective, blinded monitoring was maintained.

### Procedures

For the clinical trial, all participants were screened, enrolled, and underwent evaluation at four (two male, two female) residential addiction treatment facilities within the same organization with typical length of stays of 28 days. Senior management, therapists, nursing staff, and core services were common across all locations. The admissions process, medical and clinical assessments occurred separately for males and females at their respective facilities.

For ethical reasons and to promote feasibility, recruitment and screening occurred as quickly as possible after admission, prior to the emergence of moderate-severity opioid withdrawal signs/symptoms. Screening included obtaining informed consent, HIPAA authorization, assessment of demographics, contact data, physical exam, pregnancy testing, contraception methods, medical and drug history, and urine drug testing.

During the recruitment and informed consent process, participants were repeatedly told they could receive FDA-approved MOUD at the treatment facility instead of participating in the study. They were also told that upon discontinuation of device stimulation, or if they dropped out of the study, they could receive MOUD at any time. Participants were told that device use (active or sham treatment) would be self-administered, that they could discontinue device use at any time and for any reason, and they could receive MOUD and comfort medications.

Patients admitted for OUD, between the ages 18–65 years old, in good general health, seeking to discontinue their illicit opioid use at the treatment facility, self-reporting that they wished to become abstinent without using MOUD, were eligible to participate. All participants provided informed consent and agreed to follow study procedures and use medically accepted highly effective contraception. Prior to receiving either active or sham treatment, all participants who enrolled had to exhibit at least moderate withdrawal severity, operationally defined as a total score of >13 on the Clinical Opiate Withdrawal Scale (COWS) (38). Exclusion criteria included pregnancy or lactation, serious current psychiatric disorder (schizophrenia, bipolar) or use of neuropsychiatric medications that may overlap with NET’s proposed mechanisms of action (e.g. anxiolytics, antidepressants, anticonvulsants, sedating histamine-1-receptor antihistamines, prescription or over-the-counter stimulants), need for detoxification from alcohol or benzodiazepines, past 300-day exposure to extended-release buprenorphine, certain chronic illnesses (especially seizures), unstable medical conditions, or presence of cardiac pacemaker.

All participants received the same clinical care as all patients in the facilities, which did not include planned psychosocial interventions following discharge. At discharge, participants were advised about their increased risk for opioid overdose (due to loss of opioid tolerance during the residential stay) and were provided with naloxone and trained in how to use it. The participants were not told whether they received active or sham intervention.

### NET Device treatment

The NET Device delivers alternating current via surface electrodes placed transcranially (bilaterally) on the mastoid processes. The device delivers multiple low-amperage waveforms at controlled frequencies that vary throughout each treatment day, with no net direct current component.

In prior clinical studies, the output waveform was optimized for dynamic variations in skin impedance, electrode conductance, frequency and pulse-width related sensation, and orthogonal electrode pressure (e.g. from head pressure when sleeping), leading to improved rates of patient tolerability. Furthermore, human and animal studies, and clinical observation, have identified different electronic waveforms corresponding to subtypes of polysubstance use. These waveforms were programmed into the device at treatment entry based on each participant’s drug screen results at the time of treatment initiation. Stimulation was continuously available (except when bathing) for up to 7 days via transcutaneous electrodes of size approximately 1cm x 2cm. Stimulation output frequency varied from 4 to 3000 Hz and pulse width from 7 to 750 microseconds. Stimulation output current varied from 0 to 3.2 mA (peak) into a 15 kOhm load, and output voltage varied from 0 to 44 volts (peak to peak). Treatment was self-administered, and participants were instructed that they could control the device output intensity and duration according to perceived benefit. In prior studies, device output intensity has not been found to exhibit a significant relationship with withdrawal severity (NET Recovery Corp., unpublished data).

### Sham treatment

Sham treatment, to control for placebo effects, was designed to minimize treatment assignment recognition by the sponsor, principal investigator, independent study monitor, participants, research assistants, and treatment staff. The active and sham interventions both used the NET Device, but the sham intervention used lead wires that (although visually the same as active wires) were rendered non-conductive beforehand, preventing any electrical stimulation from being delivered to the participant.

Participants in the active and sham arms received identical instructions, equipment, electrode attachment methods and locations, and daily reviews of device operation. The apparatus presented both active- and sham-assigned participants with visual cues from the device’s “heartbeat” indicator (a blinking green light-emitting diode which indicated the device was active) during treatment. Research staff instructed each participant that the equipment was designed to be self-administered, that s/he could set the level of stimulation wherever it is comfortable, that stimulation is not always (and does not need to be) sensate, and that device use could be discontinued when the participant felt it was not providing additional benefit.

### Measures

A research assistant used a 7-day timeline follow-back (TLFB) during a weekly video call to probe for substance use. TLFB reports were specific for MOUD (methadone, buprenorphine or naltrexone, which would be expected to lower opioid use), opioids without a prescription, psychostimulants (methamphetamine without a prescription, cocaine), and sedatives. These self-reported days of use were examined as percentage of total follow-up time (84 days).

Additional measures included demographic (sex, age, race) and clinical variables (length of residential stay, primary substance use disorder), randomization group, and duration of device use (obtained from internal device statistic in 5-min intervals). Based on the observed non-normal distribution of duration of device use, this variable was dichotomized as >24 hr and <24 hr of device use leading to four groups to maximize numbers in each subgroup: Active >24 hr, Active <24 hr, Sham >24 hr, and Sham <24 hr.

### Statistical analysis

All participants who completed the trial (N = 108) were monitored during the follow-up period. Five participants who failed to complete a follow-up interview were not included in the analysis.

The primary analysis contrasted the groups randomized to active versus sham stimulation during the residential period for participation in post-discharge follow-up, completeness of data, and for outcomes using *t*-tests for continuous outcomes and *chi*-square for categorical outcomes. Subgroup analysis then dichotomized each randomization group by duration of device use (Active >24 hr, Active <24 hr, Sham >24 hr, and Sham <24 hr). When assumptions of normality were not met, nonparametric tests were used. Covariates included randomization group, duration of device use, and the demographic and clinical variables noted above.

Missing data for participants with any follow-up data were imputed as positive for substance use. In sensitivity analysis, Monte Carlo simulation was used to impute missing data based on observed means (39). Five passes were conducted with the outcomes averaged.

## Results

### Participant characteristics

A total of 108 participants (53 active NET and 55 sham; 59.3% male, 89.8% white; 71.3% fentanyl-positive, and 50.0% psychostimulant [mostly methamphetamine] UDS at screening) completed the clinical trial and were eligible for the follow-up. Five participants randomized to the active device arm were lost to follow-up (3 incarcerated, 1 lost to follow-up, and 1 protocol violation treated as unblinded compassionate care), but no one in the sham device arm. They did not differ demographically or clinically from those who completed at least one follow-up interview. **Table 1** presents their demographic and clinical characteristics at admission. **Table 2** presents demographic and clinical characteristics of the four subgroups, who completed at least one post-discharge interview. The demographics of the four subgroups did not significantly differ, as we previously found for the two randomized groups (31).

**Table 1:** Demographics and clinical characteristics of participants completing at least one timeline followback (TLFB) interview or lost to follow-up.

Measure	Completed at least one TLFB (103/108, 95.4%)		Lost to follow-up (5/108, 4.6%)	
	n or mean	Row % or SD	n or mean	Row % or SD
Demographics				
Age (years)	33.9	8.4	37.4	6.8
Male	62	60.2%	2	40.0%
Female	41	39.8%	3	60.0%
Race				
White only	92	89.3%	5	100.0%
Black only	6	5.8%	0	–
Other	5	4.9%	0	–
Hispanic or Latino Ethnicity	3	2.9%	0	–
Clinical characteristics				
Active ≥24 hours	23	22.3%	2	40.0%
Active <24 hours	25	24.3%	3	60.0%
Sham ≥24 hours	21	20.4%	0	–
Sham <24 hours	34	33.0%	0	–

**Table 2:** Demographics and clinical characteristics of participants by subgroup.

Measure	Active ≥24 hr		Active <24 hr		Sham ≥24 hr		Sham <24 hr	
	n or mean	Row % or SD	n or mean	Row % or SD	n or mean	Row % or SD	n or mean	Row % or SD
Demographics								
Completed at least one TLFB	23	22.3%	25	24.3%	21	20.4%	34	33.0%
Sex								
Male	12	52.2%	17	68.0%	9	42.9%	24	70.6%
Female	11	47.8%	8	32.0%	12	57.1%	10	29.4%
Race								
White only	21	91.3%	22	88.0%	19	90.5%	30	88.2%
Black only	1	4.3%	1	4.0%	1	4.8%	3	8.8%
Other	1	4.3%	2	8.0%	1	4.8%	1	2.9%
Hispanic or Latino Ethnicity	1	4.3%	0	0.0%	1	4.8%	1	2.9%
Clinical characteristics								
Device use (hours)	83.1	38.9	8.9	8.5	67.9	40.3	6.7	7.5

### Opioid or psychostimulant use

The mean ± 1 standard deviation (SD) percentage of days using opioids or psychostimulants (with missing values for patient reports imputed as “positive for use”) for Active (36.3 ± 34.7%) versus Sham (39.1 ± 32.6%) over the total 84-day post-discharge period did not significantly differ (*t* = .425, *p*>.10). When comparing percentage use-days for the four subgroups over the entire post-discharge period, the Active >24 hr group reported numerically but non-significantly fewer mean use-days (1.3 ± 3.4%) than Active <24 hr (4.8 ± 10.8%), Sham >24 hr (4.7 ± 15.0%), and Sham <24 hr (4.8 ± 11.2%) groups, Kruskal-Wallis *H*(3)=0.87, *p*>.05.

Rates of concurrent weekly use of opioids and psychostimulants (i.e. using both drugs at least once during the same post-discharge week) were numerically but not significantly lower in the Active group (2.4% concurrent use-weeks) than Sham (4.2%, Fisher’s exact test *p* = .192, two-tailed). Rates of concurrent weekly use were numerically but not significantly lower in the Active >24 hr group (1.0%) than Active <24 hr (3.8%, Fisher’s exact test *p* = .055, two-tailed) and significantly lower than Sham >24 hr (6.8%, Fisher’s exact test *p* = .002, two-tailed), but not significantly different than Sham <24 hr (2.6%, Fisher’s exact test *p* = .314, two-tailed).

### MOUD use

Given that post-discharge MOUD use could lead to decreased illicit opioid use, we first examined the percentage of MOUD use for the two randomized groups and four subgroups during the post-discharge period. The average percentage of days using MOUD (with missing values for patient reports imputed as “positive for use”) for Active (40.8 ± 34.2%) versus Sham (43.3 ± 33.9%) over the total 84-day post-discharge period did not significantly differ (*t* = -.367, *p* = .714). When comparing percentage use-days for the four subgroups, Active >24 hr reported numerically but non-significantly fewer mean use-days (0.7 ± 3.3%) than Active <24 hr (12.7 ± 23.6%), Sham >24 (6.0 ± 18.8%), and Sham <24 hr (9.6 ± 21.7%), Kruskal-Wallis *H*(3)=6.46, *p* = .087.

### Per-patient opioid or psychostimulant use without MOUD

The average percentage of days using opioids or psychostimulants (with missing values for patient reports imputed as “positive for use”) for Active (44.5 ± 35.3%) versus Sham (48.9 ± 34.9%) over the total 84-day post-discharge period did not significantly differ (*t* = -.635, *p* = .526). However, the percentage of use-days significantly differed by subgroup, Kruskal-Wallis *H*(3)=10.05, *p* = .012. Pairwise comparisons using Mann-Whitney *U* tests indicated that the Active >24 hr group reported significantly less-frequent use (mean=2.0 days, median=0 days) than both Active <24 hr (mean=17.5 days, median=6 days, *U* = 138.5, *z* = -3.08, *p* <.05) and Sham <24 hr (mean=14.3 days, median=1 day, *U* = 259, z=-2.15, *p* <.05). The numerical difference relative to Sham >24 hr (mean=10.8 days, median=1 day, *U* = 160, *z* = -1.92, *p* = .055) was not significant. No significant differences were found between Active <24 hr, Sham >24 hr, and Sham <24 hr groups.

With missing data imputed as use, the four subgroups did not significantly differ overall, Kruskal-Wallis test *H*(3)=5.90, *p* = .12, although the Active >24 hr group reported numerically less-frequent imputed use (mean=27.8 days, median=11 days) than Active <24 hr (mean=46.2 days, median=50 days) and Sham <24 hr (mean=41.1 days, median=44 days).

**Table 3** lists percentages of patients with zero days of opioid or psychostimulant use (complete abstinence) during the 84-day post-discharge period for the four subgroups. **Table 4** lists summary statistics for these groups. Data are presented with reported use-days and with missing values imputed as use. The percentage of participants with zero days of use was significantly higher in the Active >24 hr group (73.9%) compared to the other groups (Active <24 hr: 32.0%, Sham >24 hr: 42.9%, Sham <24 hr: 47.1%), χ^2^ = 8.97, *p* = .003.

**Table 3:** Percentage of patients in each subgroup using opioids, psychostimulants, or MOUD on 0 days (no use) and 84 days (every-day use) post-discharge.

Opioids or psychostimulants									
Group	N	Participants reporting:				Participants reporting (missing days imputed as use):			
		0 use days		84 use days		0 days		84 days	
Active ≥24 hr	23	17	(73.9%)	0	(0.0%)	5	(21.7%)	0	(0.0%)
Active <24 hr	25	16	(64.0%)	0	(0.0%)	5	(20.0%)	1	(4.0%)
Sham ≥24 hr	21	13	(61.9%)	0	(0.0%)	1	(4.8%)	1	(4.8%)
Sham <24 hr	34	24	(70.6%)	0	(0.0%)	1	(2.9%)	0	(0.0%)
MOUD									
Active ≥24 hr	23	22	(95.7%)	0	(0.0%)	6	(26.1%)	0	(0.0%)
Active <24 hr	25	14	(56.0%)	0	(0.0%)	2	(8.0%)	1	(4.0%)
Sham ≥24 hr	21	17	(81.0%)	0	(0.0%)	2	(9.5%)	0	(0.0%)
Sham <24 hr	34	23	(67.6%)	0	(0.0%)	1	(2.9%)	4	(11.8%)
Opioids, psychostimulants, or MOUD									
Active ≥24 hr	23	17	(73.9%)	0	(0.0%)	5	(21.7%)	0	(0.0%)
Active <24 hr	25	8	(32.0%)	0	(0.0%)	2	(8.0%)	2	(8.0%)
Sham ≥24 hr	21	9	(42.9%)	0	(0.0%)	1	(4.8%)	1	(4.8%)
Sham <24 hr	34	16	(47.1%)	0	(0.0%)	1	(2.9%)	4	(11.8%)

**Table 4:** Summary statistics for post-discharge days with opioid, psychostimulant, or MOUD use.

Opioids or psychostimulants								
Group	Reported use days (of 84)				Reported use days (of 84) (missing days imputed as use)			
	Mean	SD	Median	IQR	Mean	SD	Median	IQR
Active ≥24 hr	1.3	3.4	0	0-1	27.1	29.3	11	2-48
Active <24 hr	4.8	10.8	0	0-4	33.6	30.0	23	3-61
Sham ≥24 hr	4.7	15.0	0	0-2	35.1	29.6	37	8-59
Sham <24 hr	4.8	11.2	0	0-1	31.5	26.8	27	5-54
MOUD								
Active ≥24 hr	0.7	3.3	0	0-0	26.5	28.5	11	1-47
Active <24 hr	12.7	23.6	0	0-8	41.4	27.5	42	19-70
Sham ≥24 hr	6.0	18.8	0	0-0	36.5	29.0	39	5-59
Sham <24 hr	9.6	21.7	0	0-2	36.3	28.6	30	10-56
Opioids, psychostimulants, or MOUD								
Active ≥24 hr	2.0	6.2	0	0-1	27.8	29.6	11	2-49
Active <24 hr	17.5	23.5	6	0-27	46.2	27.4	50	24-72
Sham ≥24 hr	10.8	22.8	1	0-3	41.2	29.9	40	8-68
Sham <24 hr	14.4	23.6	1	0-21	41.1	29.4	44	13-64

### Group percentage of days using opioids, psychostimulants or MOUD

**Figure 1:**
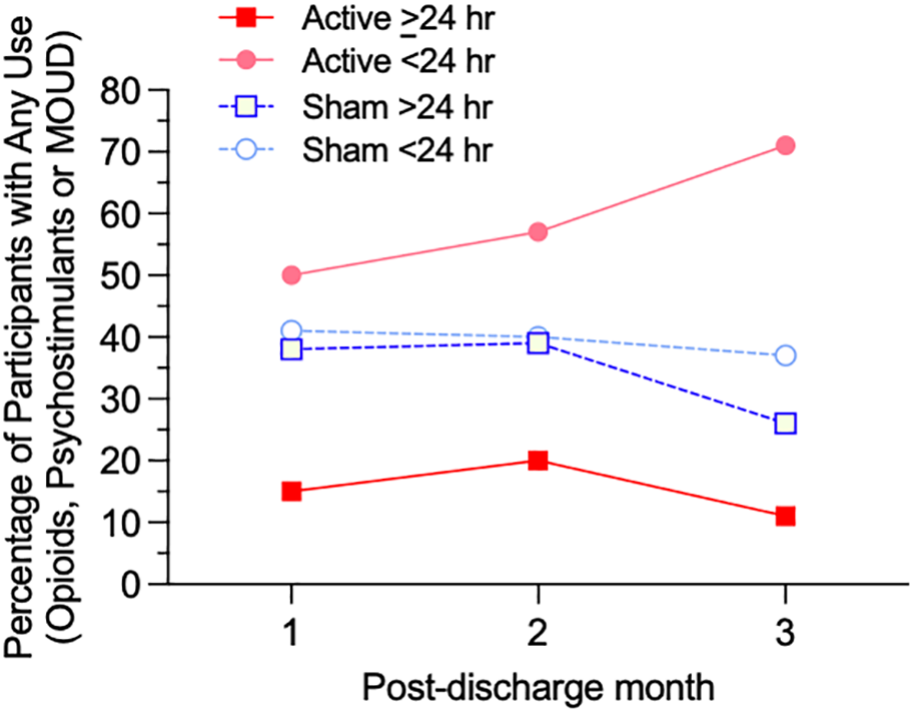
Percentage of participants with any use of opioids, psychostimulants, or MOUD for each of four subgroups (Active ≥24 hr, Active <24 hr, Sham ≥24 hr, and Sham <24 hr device utilization) for each month of the 84-day post-discharge period.

The post-discharge group percentage of person-days with illicit opioid, psychostimulants, or MOUD use (**Table 5**) was significantly lower in the Active >24 hr group (3.4% use-days) compared to Active <24 hr (31.6% use-days), Sham >24 hr (20.1% use-days), or Sham <24 hr (25.1% use-days), χ^2^ = 370.10, 172.48 and 272.25, respectively, *p* <.001. **Figure 1** presents the percentages of participants with any use of opioids, psychostimulants, or MOUD during each of the 3 months across the post-discharge period, to assess whether rates of use varied over time. Over the entire 84-day period, the proportion of participants using any illicit polysubstance or MOUD was significantly lower in the Active >24 hr group (26.1%) compared to Active <24 hr (68.0%, *p* = .004), Sham >24 hr (57.1%, *p* = .029), and Sham <24 hr (52.9%, *p* = .030, Fisher’s exact test).

**Table 5:** Person-days (percentage) using illicit opioids, illicit psychostimulants, and/or medications for opioid use disorder (MOUD) based on TLFB during the post-discharge period in active and sham device groups, stratified by duration of device use.

Group	N	Illicit opioid	Illicit psychostimulant	MOUD	Any opioid, psychostimulant or MOUD
Active total	48	115/2719 (4.2%)	70/2719 (2.6%)	333/2719 (12.2%)	483/2719 (17.8%)
Active ≥24 hr	23	19/1338 (1.4%)	17/1338 (1.3%)	16/1338 (1.2%)	46/1338 (3.4%)
Active <24 hr	25	96/1381 (7.0%)	53/1381 (3.8%)	317/1381 (23.0%)	437/1381 (31.6%)
Sham total	55	162/3073 (5.3%)	159/3073 (5.2%)	453/3073 (14.7%)	714/3073 (23.2%)
Sham ≥24 hr	21	76/1125 (6.8%)	75/1125 (6.7%)	127/1125 (11.3%)	226/1125 (20.1%)
Sham <24 hr	34	86/1948 (4.4%)	84/1948 (4.3%)	326/1948 (16.7%)	488/1948 (25.1%)

### Opioid, psychostimulant, or MOUD use (separately for each type)

**Figure 2:**
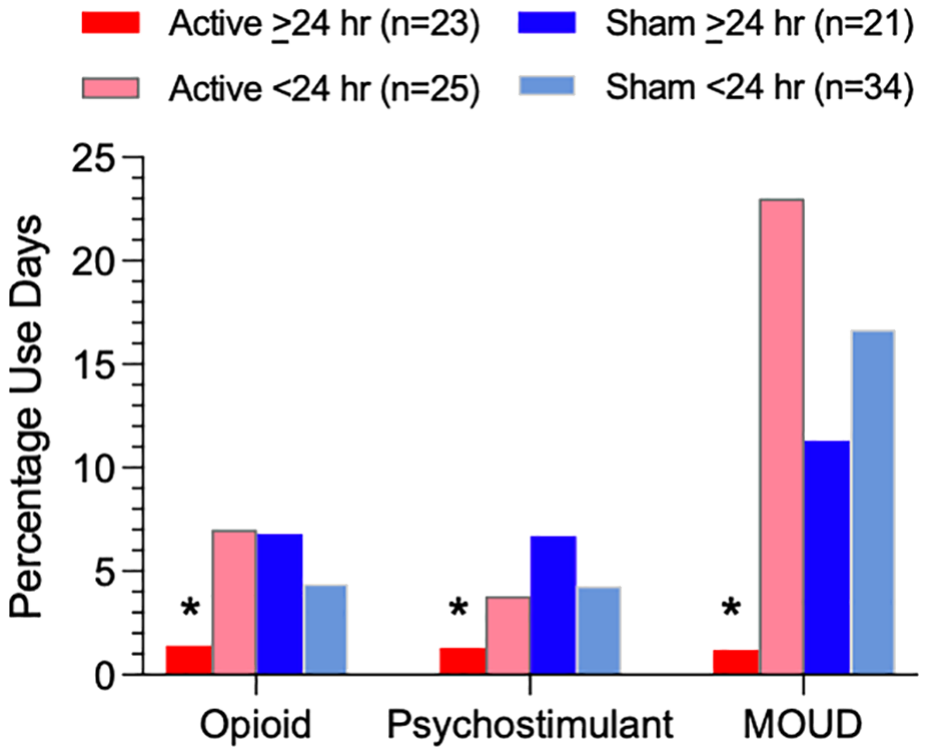
Percentage of person-days using illicit opioids, illicit psychostimulants, or MOUD based on TLFB during the post-discharge period for each of four subgroups (Active ≥24 hr, Active <24 hr, Sham ≥24 hr, and Sham <24 hr device utilization). Asterisks indicate a significant difference between the Active >24 hr group and all three other subgroups.

**Figure 2** illustrates, and **Table 5** lists, percentages of post-discharge reported person-days separately for illicit opioid, illicit psychostimulant, or MOUD use in the four groups. Rates of opioid use were significantly lower in the Active >24 hr group (1.4% use-days) compared to Active <24 hr (7.0% use-days), Sham >24 hr (6.8% use-days), or Sham <24 hr (4.4% use-days), χ^2^ = 51.33, 46.92 and 23.00, respectively, *p*s<.001, as were rates of psychostimulant use (Active >24 hr (1.3% use-days) compared to Active <24 hr (3.8% use-days), Sham >24 hr (6.7% use-days), or Sham <24 hr (4.3% use-days), χ^2^ = 17.86, 49.49 and 24.63, respectively, *p*s<.001. Rates of MOUD use were also significantly lower in the Active >24 hr group (1.2% use-days) compared to Active <24 hr (23.0% use-days), Sham >24 hr (11.3% use-days), or Sham <24 hr (16.7% use-days), χ^2^ = 299.37, 113.84 and 205.41 respectively, *p*s<.001, risk ratio from .106 to .052.

### Post-discharge patterns of use

**Figure 3:**
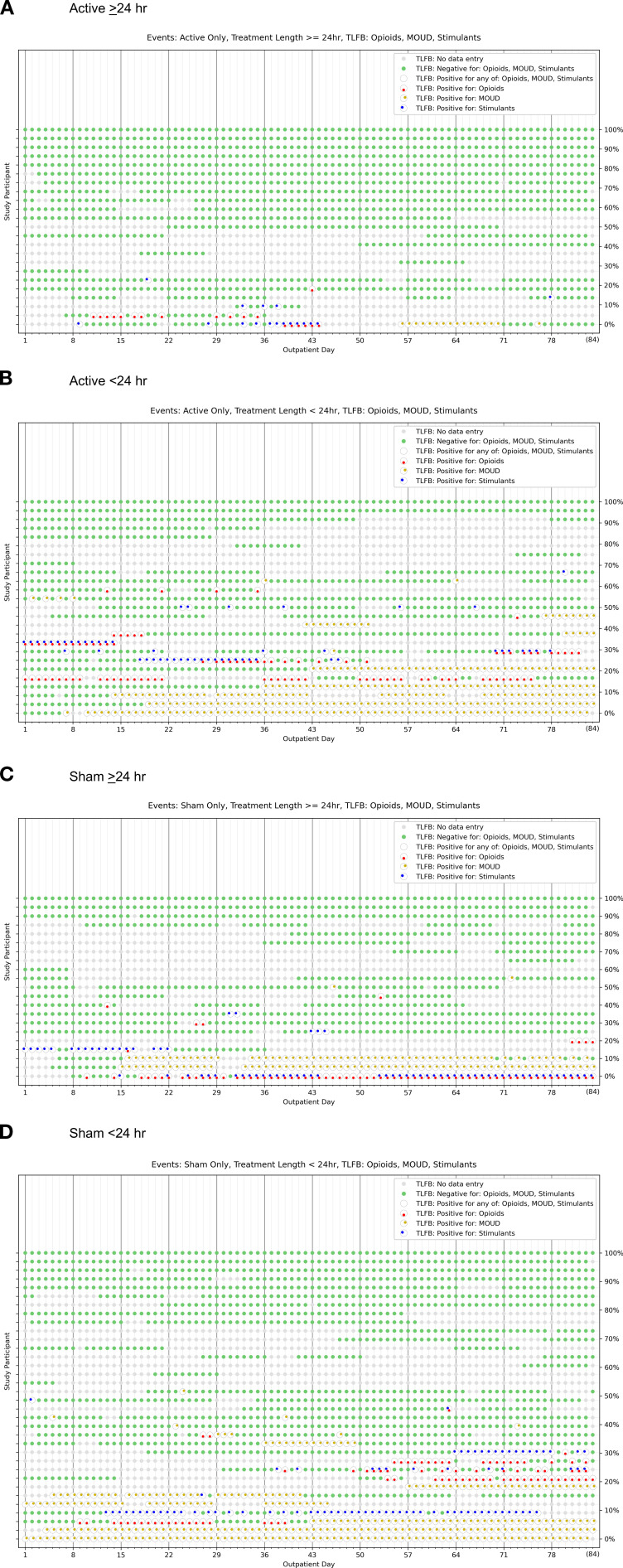
Heat maps to characterize individual-subject temporal patterns (each row) of illicit opioid, illicit psychostimulant, and MOUD use, as well as abstinence and missing data, across the 84-day post-discharge follow-up period for participants who, during the residential treatment phase, self-administered **(A)** active device stimulation >24 hours, **(B)** active device stimulation <24 hours, **(C)** sham >24 hours, and **(D)** sham <24 hours.

**Figure 3** presents heat maps that depict individual-subject temporal patterns of post-discharge daily use of opioids, psychostimulants, and MOUD, and missing data, in each of the 4 residential treatment subgroups: (A) Active >24 hr, (B) Active <24 hr, (C) Sham >24 hr, and (D) Sham <24 hr. Individuals with higher quantities of non-use reports are clustered at the top and those with higher quantities of use reports are clustered at the bottom, facilitating visual comparison between the subgroups.

### Sensitivity analyses

The Monte Carlo simulation results are consistent with analyses of reported use-days and missing data imputed as use. The Active >24 hr group had a significantly (χ^2^ = 44.84, *p* <.001) higher percentage of participants with zero days of drug use (73.9%) compared to other groups (32.0–47.1%), and no participants with 84 days of drug use.

### Completeness of follow-up data

**Figure 4:**
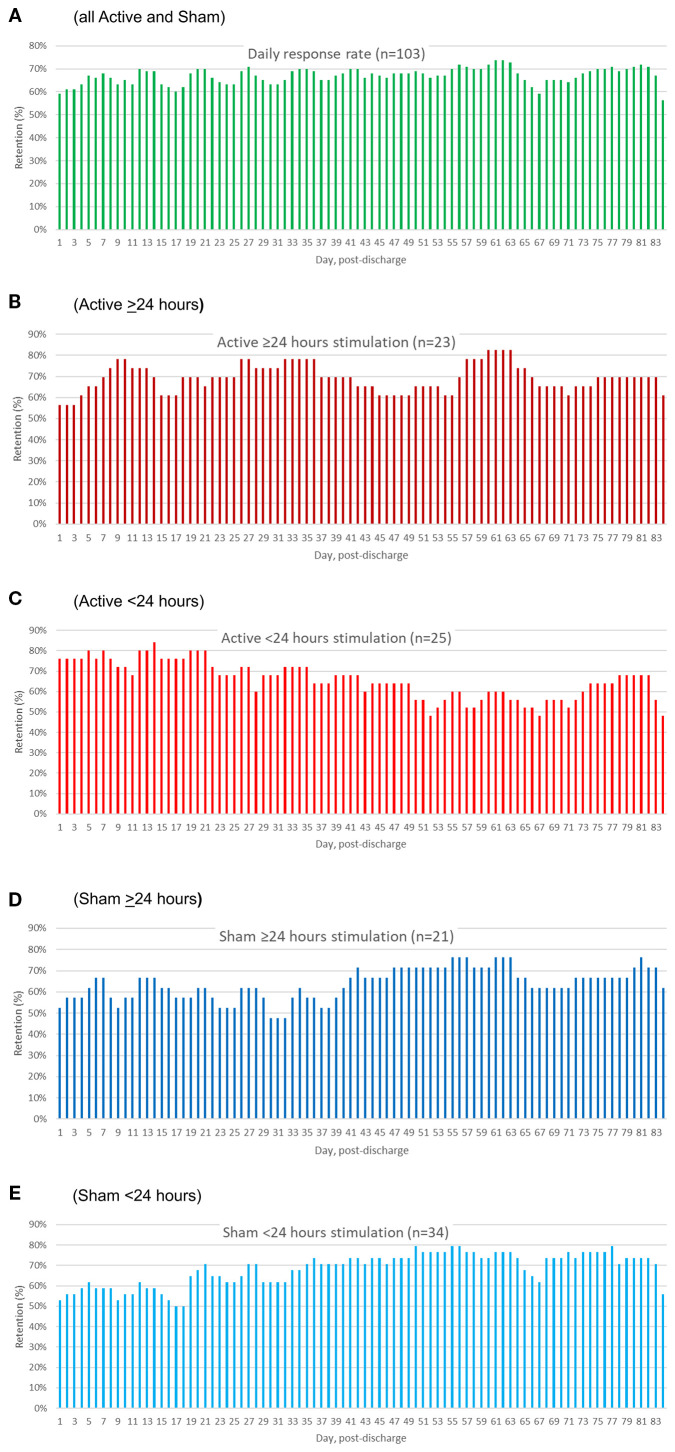
Percentage of participants reporting opioid, psychostimulant, and MOUD use data by day of follow-up. The different panels present the device utilization data separately for all patients **(A)**, Active ≥24 hr **(B)**, Active <24 hr **(C)**, Sham ≥24 hr **(D)**, and Sham <24 hr **(E)**.

**Figure 4** illustrates group-level daily percentage rates of TLFB reporting specific for all participants (4A), Active >24 hr (4B), Active <24 hr (4C), Sham >24 hr (4D) and Sham <24 hr (4E). Group-level retention did not follow a monotonically decreasing or stepwise survival function pattern, as many participants re-engaged even after periods of missing contact (56% to 74% on a given day). There were no significant differences in the number of days with no reporting between the Active and Sham groups as a whole (Mann-Whitney *U* = 1237.0, *p* = .59), or across the four groups (Kruskal-Walls *H* = .76, *p* = .69), **Table 5**.

### Demographic and clinical covariates

**Figure 5:**
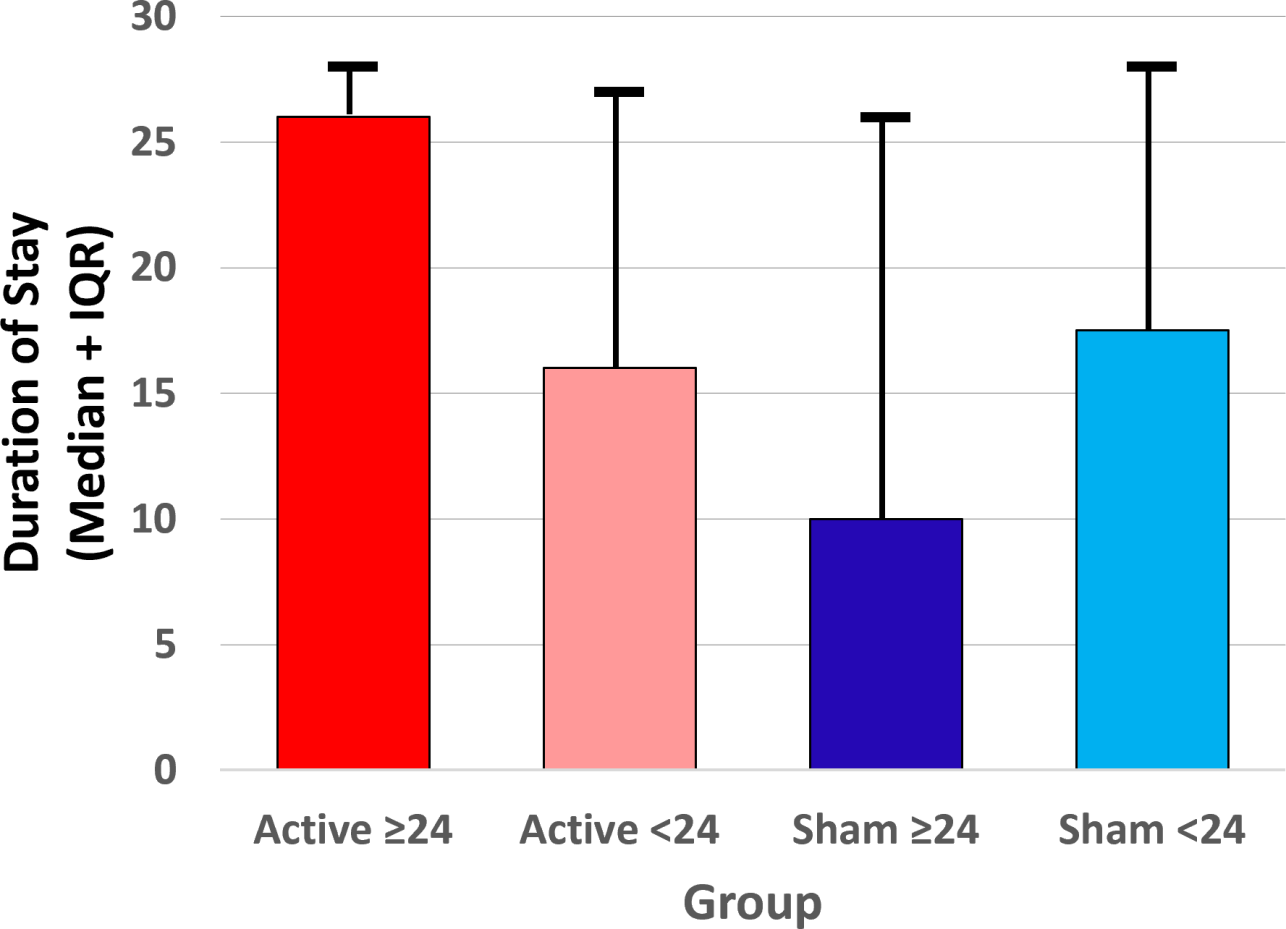
Median (interquartile range) duration of stay at the residential addiction treatment facility for each of the four subgroups (Active ≥24 hr, Active <24 hr, Sham ≥24 hr, and Sham <24 hr device utilization) prior to discharge.

The randomized groups and four subgroups did not significantly differ by sex, age, or race. Duration of stay at the residential treatment facility was analyzed using non-parametric tests due to non-normal distribution of the data. The Active group duration of stay (mean=18.5 days, median=25.5 days, IQR = 7-27) was not significantly different from Sham (mean=16.6 days, median=17 days, IQR = 6-27, *U* = 1233.5, *z* = -0.57, *p* = .57). However, the distributions significantly differed by subgroup (Kruskal-Wallis *H*(3)=49.96, *p* <.001). Pairwise comparisons using Mann-Whitney *U* tests indicated that the Active >24 hr group had significantly longer residential stays compared to Active <24 hr (*U* = 142, *p* = .003), Sham >24 hr (*U* = 114, *p* = .003), and Sham <24 hr (*U* = 267, *p* = .044) (**Figure 5**).

The correlation between residential stay duration (in 1-week bins) and whether participants used opioids, MOUD, or any of opioids, stimulants, or MOUD (binary: used vs. not used) during the post-discharge period (Spearman’s *rho* = .220, .246, and .064 respectively, *ps*>.05) was not significant.

## Discussion

The NET Device is being investigated as a therapeutic option during residential stays for persons with OUD who, given a choice, prefer not to take MOUD. The size of this subgroup of patients has not been accurately estimated in published literature but is likely a substantial minority, and is an understudied segment of the OUD population. The NET Device is not intended for all persons with OUD, given the established efficacy of MOUD and the importance of allowing patients to choose their preferred treatments. In short, this medical device option is not intended to supplant MOUD. The option to use it during inpatient stay is advantageous, because US national data indicate that residential facilities provide MOUD at very low rates (40).

We prospectively examined illicit opioid, illicit psychostimulant, and MOUD daily use rates for patients with OUD across 84 post-discharge days (12 weeks) in the Randomized, Sham-Controlled Clinical Trial to Evaluate the NET Device for Reducing Withdrawal Symptom Severity During Opioid Discontinuation (31, 36). Following discontinuation of active or sham stimulation and discharge from the residential study site, the majority (95.4%) of participants completed at least one of 12 weekly TLFB interviews.

Overall, randomization to active device stimulation did not lead to significantly fewer use-days of illicit opioids, psychostimulants or MOUD. The planned secondary analysis showed significant differences in opioid and psychostimulant use-days for active-device voluntary stimulation >24 hr compared to the other subgroups. However, participants who self-administered active stimulation >24 hr had significantly longer residential stays than those who self-administered active stimulation <24 hr or received sham. The length of residential stay might indirectly reflect the extent of psychosocial interventions received. This variable is important to consider, as participants who self-administered active stimulation longer might be more likely to engage in all forms of treatment. Our finding of no association between length of study and post-discharge illicit drug use was likely due to a modest sample size and limited variation in duration of residential stay across participants. Alternatively, greater initial opioid withdrawal suppression with NET in the active device group (31) might have improved engagement with psychosocial interventions offered during the residential stay, which could then facilitate post-discharge outcomes. Notably, participants did not receive additional planned intervention after discharge.

Relative differences in outcomes between treatment groups must be interpreted in the context of missing follow-up data. Retention in the study (indexed by the rate of daily TLFB reporting for all substances) was relatively consistent throughout the post-discharge phase. Most participants in this trial were not permanently lost to contact during the follow-up assessment period and would often reengage with post-discharge assessments. Even so, about one-third of the sample had missing TLFB data on a given day. Thus, we examined effects of different imputation strategies in our sensitivity analyses. Using our published *a priori* assumption that missing data correspond to use, which is common in substance use disorder clinical trials (41–43), absolute differences between the two randomized groups and four subgroups remained similar. Using an alternative imputation strategy of imputing data using Monte Carlo simulation, absolute use-rate estimates were lower and the relative difference between the four subgroups remained statistically significant, suggesting that the findings were robust to missing data assumptions.

This study has several limitations. First, the sample size is relatively small. Although this clinical trial was powered *a priori* to address opioid abstinence without assistance of MOUD, future studies should investigate the effectiveness of the NET Device in larger and more heterogeneous patient samples outside a single geographical area. Second, the finding that self-administering active stimulation >24 hr (relative to active <24 hr or sham) was associated with less post-discharge substance use emerged from a planned secondary analysis that should be replicated in an independent sample. The reasons behind this finding are unclear. For instance, different durations of device use might be due to the policy at this treatment center that all patients must wait 24 hr before they can qualify to receive MOUD (setting effect). Unmeasured individual differences could also contribute to this outcome. The finding might reflect that patients had effective device-related acute suppression of opioid withdrawal and benefited from residential psychosocial interventions, leading to better post-discharge outcomes. However, short-term use of the NET Device to suppress opioid withdrawal during residential treatment does not provide any guarantee of decreasing relapse. Third, the neurobiological mechanisms that underlie any possible relationship between active device use and rates of illicit substance use remain to be determined. Lastly, the follow-up period was limited to 3 months, thus, longer-term outcomes remain unclear. We are now conducting a follow-up study to determine outcomes at 2 or more years post-study, which will provide useful information on the durability of treatment effects.

In conclusion, participants who self-administered active NET Device stimulation >24 hr during residential treatment reported during the 84-day post-discharge period significantly fewer use-days of illicit opioids or psychostimulants without relying on MOUD, compared to other participants. Taken together, the NET Device may be appropriate and useful for individuals with OUD who are in residential treatment and do not want to pursue recovery with medications, but these findings need to be replicated.

## Data Availability

The datasets presented in this article are not readily available because the data are proprietary to NET Recovery Corp. Requests to access the datasets should be directed to joe.winston@netrecovery.net.
